# Cost structure in specialist mental healthcare: what are the main drivers of the most expensive episodes?

**DOI:** 10.1186/s13033-023-00606-6

**Published:** 2023-11-09

**Authors:** Yeujin Ki, Andrew Athan McAleavey, Tron Anders Moger, Christian Moltu

**Affiliations:** 1 Department of Research and Innovation, Helse Førde, Førde, Norway; 2 Department of Psychiatry, Helse Førde, Førde, Norway; 3 Department of Health and Caring Sciences, Western Norway University of Applied Science, Bergen, Norway; 4 Department of Health Management and Health Economics, University of Oslo, Oslo, Norway; 5 Section of Medical Statistics, Faculty of Medicine, University of Oslo, Oslo, Norway

**Keywords:** Specialist mental healthcare, Cost per patient (CPP), Kost per pasient (KPP), Economic analysis, Hospital administration

## Abstract

**Background:**

Mental disorders are one of the costliest conditions to treat in Norway, and research into the costs of specialist mental healthcare are needed. The purpose of this article is to present a cost structure and to investigate the variables that have the greatest impact on high-cost episodes.

**Methods:**

Patient-level cost data and clinic information during 2018–2021 were analyzed (N = 180,220). Cost structure was examined using two accounting approaches. A generalized linear model was used to explain major cost drivers of the 1%, 5%, and 10% most expensive episodes, adjusting for patients’ demographic characteristics [gender, age], clinical factors [length of stay (LOS), admission type, care type, diagnosis], and administrative information [number of planned consultations, first hospital visits, interval between two hospital episode].

**Results:**

One percent of episodes utilized 57% of total resources. Labor costs accounted for 87% of total costs. The more expensive an episode was, the greater the ratio of the inpatient (ward) cost was. Among the top-10%, 5%, and 1% most expensive groups, ward costs accounted for, respectively, 89%, 93%, and 99% of the total cost, whereas the overall average was 67%. Longer LOS, ambulatory services, surgical interventions, organic disorders, and schizophrenia were identified as the major cost drivers of the total cost, in general. In particular, LOS, ambulatory services, and schizophrenia were the factors that increased costs in expensive subgroups. The “first hospital visit” and “a very short hospital re-visit” were associated with a cost increase, whereas “the number of planned consultations” was associated with a cost decrease.

**Conclusions:**

The specialist mental healthcare division has a unique cost structure. Given that resources are utilized intensively at the early stage of care, improving the initial flow of hospital care can contribute to efficient resource utilization. Our study found empirical evidence that planned outpatient consultations may be associated with a reduced health care burden in the long-term.

## Background

Mental disorders are the costliest conditions to treat in Norway [1], both in terms of health care expenditures and gross societal losses [2]. Consequently, the financial burden of mental illness has been studied from a various of angles, including societal perspective [3] and healthcare provider perspective [1]. In Norway, the Norwegian Directorate of Health provides annual health care service cost information based on data submitted by healthcare providers [4]. Since these cost data are collected and utilized primarily for management purposes in Norway, such as reimbursement and productivity comparison, they are more frequently employed for macro-level (regions and institutions) analysis. Perhaps because individual direct payments are relatively low in Norway, micro-level data (individual cost data) are relatively less frequently analyzed than in countries with high out-of-pocket expenses, where they must be analyzed for billing purposes. However, individual cost data can have major implications for systems and individuals, regardless of the direct payment sources. Moreover, cost studies are essential for providing high-value services in a sustainable manner. In order to assess whether an intervention provides good value, it is necessary to access intervention’s cost information as well as their clinical benefits and risks [5]. However, research indicates that general cost awareness is low in many countries [6–9], which necessitates enhanced access to cost information and training to be able to make more efficient and optimal decisions [10, 11]. As an example, in 2021, one Norwegian municipality proposed a 600,000 USD (“hoping to convince politicians”) as initial funding for a pilot program to introduce early care for substance-abuse patients [12]. A government document about the pilot program had reported a 33% reduction in hospital stays [13]. In a situation such this, people can make effective arguments and decisions about what to do if they are aware of the current scale of resources utilized by substance-abuse patients and the financial impact of a 33% reduction in days of hospitalization. Just a 21% reduction in the hospital stays of the one substance-abuse patient who consumed the most resources in 2021 would result in a savings of 620,000 USD, which shows that the suggested amount of initial funding would be negligible in light of its overall effect. Increased availability of cost studies and data will facilitate policy discussions based on the evidence.

Historically, cost studies were conducted in order to compare hospital reimbursements with actual expenses [14–16]. In the early phase of cost studies in clinical settings, hospital staff manually recorded patients’ resource utilization [17]. This method, however, had a great risk of losing precision. Due to the development of an automated hospital administration system, cost data can now be gathered directly with improved accuracy. One of the systems that has potential for the field of healthcare cost studies is the Cost Per Patient (CPP). The CPP is a patient-level cost-calculation model that is designed to collect data and demonstrate how resources are used by each patient during each hospital visit [18]. The concept of a patient-level cost-tracking system is used in various countries under different names. In England, the system is called the Patient-Level Information and Costing System (PLICS), which was implemented by the National Health Service (NHS) in 2015 [5]. Sweden calls its system Kostnad Per Pasient (KPP), which has been in operation since the early 1990s [6]. Though Germany’s healthcare system is different from the NHS model used in countries such as Sweden and England, Germany’s “Instituts für das Entgeltsystem im krankenhaus (InEK)” was established in 2001 to manage a comprehensive pricing system [7]. One of the organization’s main tasks is calculating patient-level costs [8]. One of the advantages of cost data derived from the CPP is that it is based on local “bottom–up” costing techniques where the costs of episodes reflect the actual expenditures required to provide care. Domestic cost studies can provide the most accurate information because the specific arrangement of national healthcare service provision that determines how and where costs are likely to incur vary from country to country.

This study aimed to analyze the cost structure of specialized mental healthcare and identify the main cost drivers of expensive episodes. It would be advantageous for clinicians to have an understanding of the hospital's resource utilization so that they can voice their professional opinions also on administrative decisions based on costs.

## Materials and methods

### Data source

The data were collected from, Helse Førde, a regional health enterprises in Norway. Helse Førde is part of Western Norway Regional Health Authority (Helse Vest). Helse Førde serves approximately 109,000 inhabitants in eighteen municipalities, as of 2023. The data were extracted from the CPP system from January 1, 2018 to December 31, 2021. The inclusion criteria were cost records of patients of any age who received inpatient or outpatient treatment in the specialized mental healthcare service during this time period. Once patients register their social security number and the name of a hospital system, the cost is calculated along with current-stay information, such as date of arrival and time of discharge. Each hospital visit is recorded as a single episode with one diagnosis-related group (DRG) code (e.g., episode 1: a one-hour outpatient session due to depressive symptoms, episode 2: a hospital stay of ten days coded with the diagnosis of schizophrenia). One hundred and twenty three hospital episodes of incorrect grouping and double records were excluded from the analyses. Twenty-three episodes of unknown admission type were also excluded. In summary, 180,220 episodes, which represented 99.9% of all actual episodes during the inclusion period, were analyzed, making the included data excellent in terms of their completeness in mental health service research.

### Study design

**Fig. 1:**
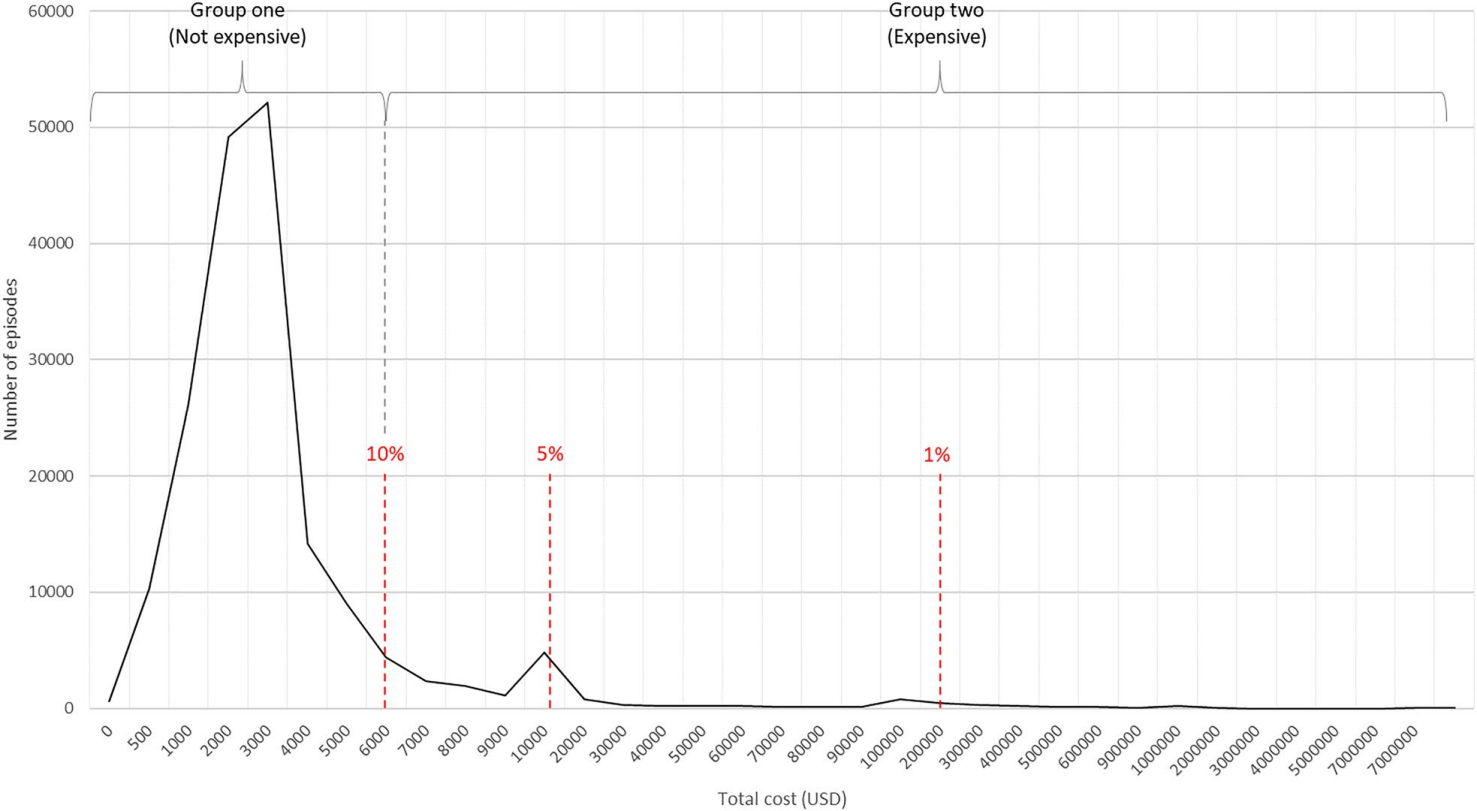
Illustration of the two-part model: cost distribution of 180,220 episodes

This was a cross-sectional retrospective study on hospitals’ patient-level cost-data of the specialist mental healthcare division. This study adopted the classic two-part model for the cost analysis [19, 20]. The idea of grouping episodes by two categories (expensive and not) is that the episodes in hospitals are frequently a mixture of two types. Some patients visit the hospital for minor clinical needs, such as assessment and advice, while others require expensive treatment, such as an operation and long-term hospitalization. The former episodes are represented by values close to zero in the right-skewed cost distribution graph shown in Fig. 1, whereas the latter group is represented in the heavy right tail. We used three different cut-off points of 1%, 5%, and 10% of total costs in line with international studies [21]; thus, those in and above the 99th, 95th, and 90th percentiles of the cost distribution were defined as expensive groups. We examined the cost structure and cost drivers of these expensive groups based on this two-part model.

Cost structure was analyzed from two simplified perspectives; the traditional perspective that entails the basic concepts of general accounting [22] and a relatively recent perspective (introduced in the 2000s) based on a hospital’s activity [23, 24]. The latter has been recommended for hospital cost accounting [25] as a ‘time-driven activity-based costing’ (henceforth, activity-based costing). With the traditional perspective, costs were classified as direct and indirect costs. For the activity-based perspective, costs were classified according to a hospital’s seven main activities: ambulatory service, intensive care, operations, anesthesia, radiology, and outpatient and inpatient care. Each cost category consisted of eight sub-costs elements, including direct labor costs (clinicians and other healthcare professionals), direct consumable costs (medicines, main consumables, and other consumables), and indirect costs (capital costs and other overhead costs) (see Appendix 1).

Multivariate generalized linear models (GLMs) with ten independent variables were used to identify cost drivers: two demographic variables [gender and age], four clinical variables [length of stay (LOS), admission type, care type, and diagnostic-related group (DRG)] and three administrative variables [number of planned consultations, first hospital visits, and interval between two hospital episodes (interval since last hospital episode)]. This study included basic patient information that has been reported to be associated with high healthcare costs, such as age, gender, DRG, and LOS [26–28]. We also analyzed new variables that were available via the CPP system to see if they affected total costs. The three new variables we included were “first hospital visits,” “interval between two episodes,” and “number of planned consultations.” The first hospital visit variable refers to the first visit registered during a follow-up period of four-years, which tended to be higher in the expensive groups than the average. The interval between two episodes was included to determine whether episodes that were too close together or too far apart may affect costs possibly because they represent early discharge or dropout. The number of planned consultations was the total number of planned psychiatric-/psychotherapeutics consultations performed by both psychologists and psychiatrists. This variable was used to determine whether planned consultations affected both total costs and average costs.

Age and gender were recorded at the time of inclusion. Seventy-three DRG codes were condensed into nineteen main categories based on professional judgment (see Appendix 2). Prior to analysis, some diagnostic categories were replaced with “Anonymous” to maintain anonymity if there were less than five patients in a diagnostic group within the same age group. The DRG category of “Not specified” refers to episodes that did not contain diagnostic information, such as “family-centered outpatient services.” Surgical intervention refers to any surgical procedure performed in a specialist mental healthcare division, as defined by the hospital, such as anesthesia involved in electroconvulsive therapy. LOS was classified into five categories: day treatment (outpatient within 1 day); less than one-week (1–7 days); less than a month (8–30 days); less than three months (31–90 days); and more than three months and less than one year (91–365 days).

Admission type and care type were used to distinguish the types of hospital visits, such as acute/planned visits and outpatient/inpatient service. The total cost of each episode was used as the dependent variable in order to identify cost drivers affecting the total consumption of resources. Cost per day (total cost/LOS) was also used as a dependent variable to eliminate the effect of accumulated hospital days.

### Statistical analysis

Descriptive statistics were used the estimate the mean and 95% confidence intervals of total healthcare services costs. The GLM was used because the dependent variable “cost” was highly skewed to the right and it did not have negative values. Although there is no single optimal or dominant model for resource utilization and cost analysis in healthcare [29], the GLM with a log transformation and the Gamma distribution has been recommended for cost analysis because of its better performance in estimating population means and its realistic description of cost data [30–32]. We tested six different GLM models on our dataset, with log and square root transformations, and Gaussian, Poisson, and Gamma distributions. We compared the Akaike information criterion (AIC) [33] and the Bayesian information criterion (BIC) [34] of each model. The model with the lowest AIC and BIC in our dataset was the square root transformation with a Gamma distribution. However, the differences between the log transformation with the Gamma distribution model were not significant based on the AIC and BIC values, and as a log transformation is more widely used in this field, we opted to use the log distribution with a Gamma distribution model to facilitate comparisons with other studies. Independent variables that were statistically significant in the bivariate analyses were included in the multivariate model; a 5% significance level was used throughout. The average marginal and incremental effects of each variable were calculated in United States dollars (USD). Using the average exchange rate from 2021 to 2022, 10 NOK was converted to 1 USD. The data were analyzed in STATA SE version 17.

## Results

### Patients’ background characteristics

Table 1 displays the background characteristics of the patients. Compared to all the episodes, the expensive groups had a higher proportion of male patients. The adolescent group had the largest proportion of all episodes, whereas age was more evenly distributed across the expensive subgroups. The expensive groups had a higher proportion of “acute admissions,” “first hospital visits,” and “anonymous” categories in diagnosis.

**Table 1:** Patients’ background characteristics

Variable	All episodes (n = 180 220)		1% expensive episode (n = 1 802)		5% expensive episode (n = 9 139)		10% expensive episode (n = 18 010)	
**Resource use**								
**Total**, K USD (%)	188,489	100%	107,733	57%	133,916	71%	140,696	75%
**Average**, per episode per day, USD (SD)	385	360	1537	577	1613	701	1195	660
**Male**, N (%)	78,780	44%	1007	56%	4561	50%	9341	52%
**Age**								
0–9	15,545	9%	0	0%	553	6%	2038	11%
10–19	57,441	32%	210	12%	1807	20%	4769	26%
20–29	33,501	19%	291	16%	1791	20%	3321	18%
30–39	28,646	16%	343	19%	1608	18%	2762	15%
40–49	21,402	12%	314	17%	1465	16%	2402	13%
50–59	14,217	8%	292	16%	987	11%	1453	8%
60–69	5979	3%	192	11%	548	6%	726	4%
70 +	3489	2%	160	9%	380	4%	539	3%
**Length of stay (LOS)**								
Care within 1 day	176,210	98%	0	0%	5160	56%	14,013	52%
1–7 days	1,559	1%	13	1%	1539	17%	1554	6%
8–30 days	1,557	1%	940	52%	1551	17%	1553	6%
31–90 days	706	0%	675	37%	705	8%	706	3%
91–365 days	188	0%	174	10%	184	2%	184	1%
**LOS**, mean (SD)	0.57	0.01	43	0.95	10	0.26	5	0.14
LOS Care within 1 day, mean (%)	0.04	98%			0.14	56%	0.10	78%
LOS More than one night, mean (%)	23	2%	43	100%	23	44%	23	22%
**First hospital visit**								
Yes	10,363	6%	293	16%	1039	11%	1646	9%
No	169,857	94%	1509	84%	8100	89%	16,364	91%
**Interval since last hospital episode**								
Very short re-visit (-3 days)	17,482	10%	321	18%	1739	19%	2642	15%
3–7 days	37,083	21%	297	16%	1737	19%	3234	18%
7–30 days	74,892	42%	373	21%	2411	26%	5721	32%
30–90 days	24,854	14%	258	14%	1147	13%	2473	14%
90 days -	7452	4%	216	12%	704	8%	1170	6%
No previous history or anonymous	18,457	10%	337	19%	1401	15%	2770	15%
**Admission type**, N (%)								
Planned	177,607	99%	1185	66%	7132	78%	15,904	88%
Acute	2613	1%	617	34%	2007	22%	2106	12%
**Care type**, N (%)								
Outpatient consultation	160,253	89%	0	0%	4354	48%	10,753	60%
Ambulatory outpatient consultation	7396	4%	0	0%	427	5%	1485	8%
Ambulatory other services	8395	5%	0	0%	357	4%	1674	9%
Inpatient	3954	2%	1668	93%	3809	42%	3888	22%
Ambulatory inpatient	89	0.0%	48	3%	87	1%	89	0.5%
Surgical intervention	133	0.1%	86	5%	105	1%	121	1%
**Number of planned consultation**, mean (SD)	15	0.1	27	1.0	24	0.5	22	0.4
**Diagnose**, N (%)								
Emotional	26,299	15%	0	0%	475	5%	1407	8%
Depressive disorder	14,599	8%	0	0%	158	2%	556	3%
Trauma	15,600	9%	0	0%	123	1%	469	3%
Anxiety	11,967	7%	52	3%	283	3%	630	3%
Schizophrenia	3782	2%	94	5%	729	8%	1071	6%
Other psychoses	2193	1%	291	16%	712	8%	800	4%
Bipolar	4763	3%	110	6%	323	4%	548	3%
ADHD	4508	3%	0	0%	69	1%	236	1%
Geriatric	3451	2%	0	0%	69	1%	237	1%
Eating disorder	2990	2%	15	1%	51	1%	112	1%
Personality disorder	2130	1%	15	1%	143	2%	197	1%
OCD	1327	1%	0	0%	96	1%	156	1%
Substance abuse	5309	3%	350	19%	698	8%	907	5%
Development disorder	540	0.3%	0	0%	13	0.1%	34	0.2%
Stress	362	0.2%	109	6%	361	4%	361	2%
Organic disorder	15	0.0%	6	0.3%	15	0.2%	15	0.1%
Neuropsychiatric	8	0.0%	4	0.2%	8	0.1%	8	0.0%
Not specified	71,926	40%	43	2%	2815	31%	7882	44%
Anonymous	8451	5%	713	40%	1998	22%	2384	13%

### Cost structure of specialist mental healthcare divisions

Table 2 presents the cost structure of specialist mental healthcare divisions by the expensive and non-expensive groups. High labor (personnel) costs were observed, and labor costs were estimated to account for approximately 87% of total expenditures. Compared to the previous SAMDATA report by the Norwegian Directorate of Health, outpatient consultation and bed-day costs are comparable (see Appendix 3) [4]. From an accounting perspective, direct and indirect costs were constant across the expensive and non-expensive episodes. However, the activity-based hospital cost structures differed between the expensive and inexpensive groups. The results showed that the more expensive an episode was, the higher the proportion of inpatient care costs were (ward cost). Inpatient care costs accounted for 99% of the costs of the 1% most expensive episodes, 93% of the costs of the 5% most expensive episodes, and 88% of the costs of the 10% most expensive episodes. In contrast, inpatient care costs generally accounted, on average, for only 66% of total costs. Another feature that stood out was that a small number of episodes consumed the majority of available resources, with 57% (USD 107.7 million out of 188 million, see Table 2) of hospital resources being allocated to 1% of hospital episodes.

**Table 2:** Cost structure of specialist mental healthcare divisions

(K USD)														
Group Categories	Traditional perspective					Activity-based perspective								
	Direct			Indirect	Total sum	Ward	Policlinic	Ambulant	Radiology	Surgery	Anesthesia	Intensive	Medicines	Total sum
		Personnel	Material											
Expensive 1% (1 802 episodes)	94,067	92,885	1181	13,667	107,733	106,829	589	30	105	122	56	1	0.05	107,733
	87%	86%	1%	13%	100%	99%	1%	0.0%	0.1%	0.1%	0.1%	0.0%	0.0%	100%
Expensive 5% (9 139 episodes)	117,100	115,733	1367	16,816	133,916	124,329	8036	1205	144	139	62	1	0.09	133,916
	87%	86%	1%	13%	100%	93%	6%	1%	0.1%	0.1%	0.0%	0.0%	0.0%	100%
Expensive 10% (18 010 episodes)	123,088	121,704	1384	17,608	140,696	124,396	12,898	3006	185	144	64	1	0.31	140,696
	87%	87%	1%	13%	100%	88%	9%	2%	0.1%	0.1%	0.0%	0.0%	0.0%	100%
Least espensive case 90% (162 210 episodes)	42,362	42,269	93	5431	47,793	21	42,305	5330	132	2	0.9	-	3	47,793
	89%	88%	0%	11%	100%	0%	89%	11%	0.3%	0.0%	0.0%	0.0%	0.0%	100%
All episodes (180 220 episodes)	165,450	163,973	1477	23,039	188,489	124,416	55,203	8336	318	146	65	1	3	188,489
	88%	87%	1%	12%	100%	66%	29%	4%	0.2%	0.1%	0.0%	0.0%	0.0%	100%

### General analysis: what are the main cost drivers?

The GLM results of the general analysis are shown in Table 3. Since we used a multivariate model, each variable must be interpreted in the context of controlling for the other variables to estimate its unique contribution to costs. LOS was found to have the greatest impact on the total increase in cost, among all the statistically significant variables. The total cost increased as LOS increased (USD 2,415 ~ 65,088), but the average cost (calculated by dividing the total cost by the LOS) of short episodes (LOS 1-7) had the greatest impact on the cost increase (USD 628). The variable that made the second largest contribution to the total cost was type of care. Specifically, surgical interventions had the largest impact on both the total and average costs. The effect of ambulatory service on the cost increase differed depending on whether it led to a hospitalization or not. The first hospital visit, a very short or long hospital re-visit (within 3 days and after more than 90 days), and younger age (0–9 years old) were also associated with an increase in cost.

**Table 3:** Results of the GLM for the general analysis

All episodes (n = 180 220)	Total cost per episode				Average cost per day			
	Cost change (USD)	p-value		95% CI	Cost change (USD)	p-value		95% CI
**Gender** [Female]*								
Male	9	0.04	0	17	3	0.04	0	6
**Age** [20–29]*								
0–9	153	0.00	133	174	53	0.00	46	60
10–19	48	0.00	35	60	16	0.00	12	20
30–39	− 37	0.00	− 51	− 23	− 14	0.00	− 19	− 9
40–49	− 11	0.16	− 26	4	− 4	0.11	− 10	1
50–59	− 59	0.00	− 76	− 42	− 22	0.00	− 28	− 16
60–69	− 226	0.00	− 250	− 203	− 83	0.00	− 90	− 75
70 +	− 298	0.00	− 330	− 267	− 107	0.00	− 117	− 96
**Length of stay (LOS)** [Care within 1 day]*								
1–7 days	2415	0.00	2091	2740	628	0.00	524	732
8–30 days	11,234	0.00	9866	12,602	441	0.00	353	529
31–90 days	30,940	0.00	27,023	34,856	375	0.00	288	461
91–365 days	65,088	0.00	54,629	75,546	193	0.00	107	279
**Number of planned consultations**	− 0.5	0.00	− 0.6	− 0.4	− 0.2	0.00	− 0.2	− 0.1
**First hospital visit** [Not a first visit]*	217	0.00	187	247	80	0.00	69	91
**Interval since last hospital episode** [7–30 days]*								
Very short re-visit (-3 days)	166	0.00	149	182	61	0.00	55	67
3–7 days	19	0.00	8	29	7	0.00	3	11
30–90 days	13	0.03	1	25	5	0.04	0	9
90 days -	113	0.00	91	135	41	0.00	33	49
No previous history or anonymous	− 6	0.53	− 26	13	− 2	0.53	− 9	5
**Admission type**, N (%) [Planned]*								
Acute	− 151	0.00	− 194	− 108	− 25	0.00	− 41	− 10
**Care type**, N (%) [Outpatient consultation]*								
Ambulatory outpatient consultation	284	0.00	260	309	157	0.00	147	167
Ambulatory other services	374	0.00	345	403	206	0.00	195	216
Inpatient	812	0.00	683	942	303	0.00	234	372
Ambulatory inpatient	1175	0.00	838	1512	359	0.00	220	498
Surgical intervention	1160	0.00	890	1429	450	0.00	323	576
**Diagnose**, N (%) [Depressive disorder]*								
Emotional	65	0.00	49	82	26	0.00	19	32
Trauma	23	0.01	6	41	9	0.01	2	16
Anxiety	13	0.17	− 6	32	6	0.14	− 2	13
Schizophrenia	517	0.00	474	560	199	0.00	183	215
Other psychoses	201	0.00	160	243	75	0.00	59	91
Bipolar	76	0.00	49	103	28	0.00	18	39
ADHD	− 45	0.00	− 70	− 19	− 17	0.00	− 27	− 7
Geriatric	295	0.00	246	343	118	0.00	99	136
Eating disorder	− 54	0.00	− 83	− 25	− 21	0.00	− 33	− 10
Personality disorder	70	0.00	32	108	27	0.00	12	41
OCD	276	0.00	221	332	109	0.00	88	130
Substance abuse	2	0.88	− 23	27	1	0.82	− 9	11
Development disorder	30	0.39	− 38	99	12	0.37	− 15	39
Stress	71	0.13	− 21	162	95	0.00	53	136
Organic disorder	675	0.05	5	1346	268	0.05	6	530
Neuropsychiatric	450	0.26	− 339	1239	56	0.65	− 183	295
Not specified	− 39	0.00	− 53	− 24	− 15	0.00	− 20	− 9
Anonymous	182	0.00	158	207	73	0.00	63	82

* Reference categories in brackets

### Expensive episodes analysis: what are the main cost drivers?

**Fig. 2:**
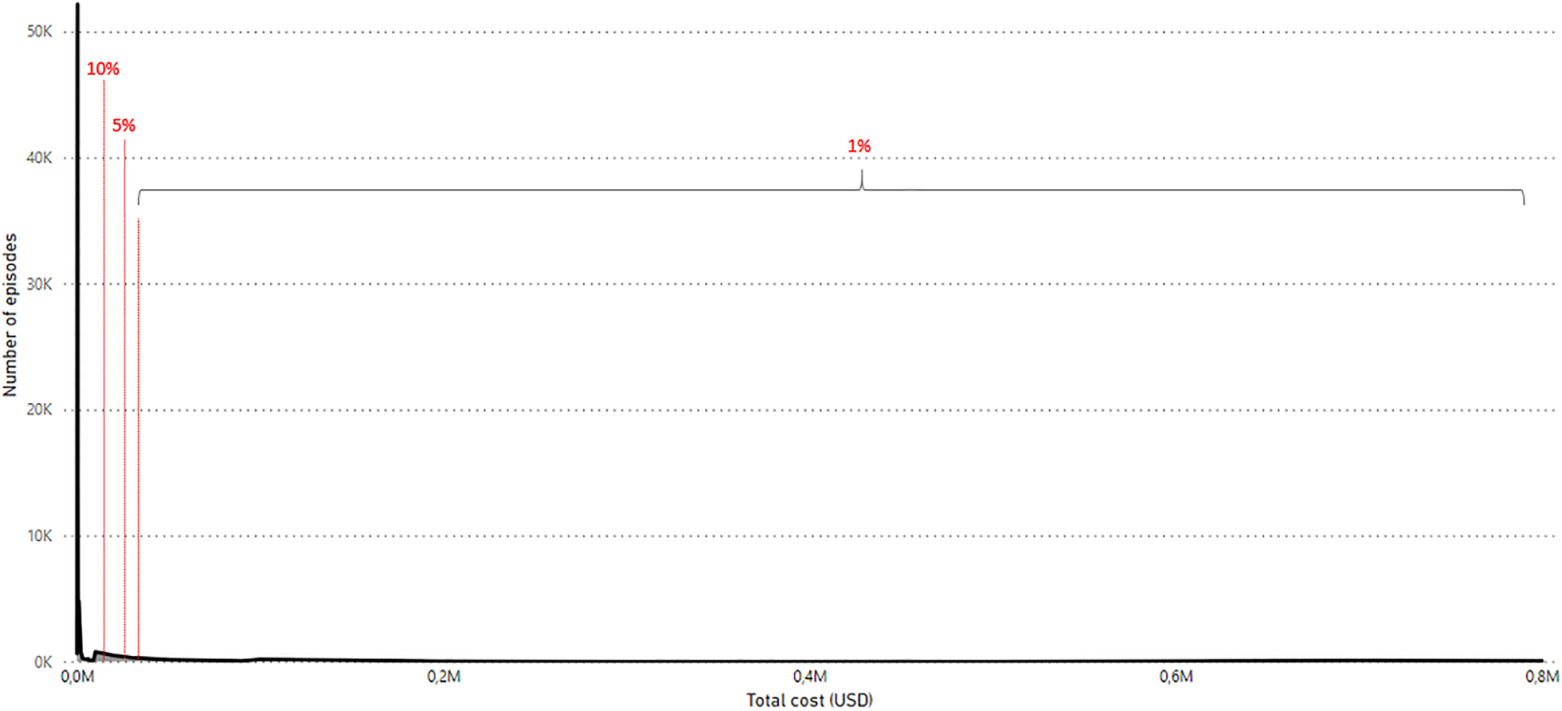
Cost distribution of 180,220 episodes in the actual USD scale

Although there were inconsistencies between the subgroups, the cost drivers of expensive episodes did not differ significantly from those in the general analysis. Table 4 illustrates the results of the GLM of high-cost episode groups. LOS was found to have the greatest impact on total expenditure in all the groups, and was similar to the general analysis; i.e., the average cost per day decreased as the LOS increased (except for the 1% group). The 1% most expensive group showed a similar tendency in which a shorter LOS decreased the average cost (USD 973 → 822 → 656), but this did not apply to hospitalizations longer than three months (USD 827). As indicated by the highly skewed cost curve, 1% of the episodes were extremely expensive, even compared to the 5% and 10% episodes (Fig. 2); consequently, the effect of LOS on the 1% group appears to be quite distinct from that of some variables. The variables that were statistically significant for the 5% and 10% groups (e.g., as sex, a very short re-visit, and the number of planned consultations) were not significant for the 1% group, possibly because of condition severity in the 1% group or its smaller sample size, compared to the other groups.

**Table 4:** Results of GLM for the 1%, 5%, and 10% expensive groups

		Total cost per episode						Average cost per day					
		1% (1 802)		5% (9 139)		10% (18 010)		1% (1 888)		5% (9 012)		10% (18 420)	
		Cost change (USD)	p-value	Cost change (USD)	p-value	Cost change (USD)	p-value	Cost change (USD)	p-value	Cost change (USD)	p-value	Cost change (USD)	p-value
**Gender** [Female]													
Male		237	0.85	336	0.04	260	0.00	24	0.52	39	0.01	36	0.00
**Age** [20–29]													
0–9				− 626	0.06	− 1058	0.00	− 133	0.14	− 144	0.00	− 211	0.00
10–19		35,270	0.00	2310	0.00	− 216	0.02	257	0.00	153	0.00	− 90	0.00
30–39		− 207	0.91	719	0.00	253	0.02	102	0.07	19	0.38	13	0.41
40–49		491	0.78	1096	0.00	386	0.00	97	0.09	70	0.00	31	0.06
50–59		680	0.71	466	0.10	180	0.18	125	0.09	− 33	0.18	− 13	0.50
60–69		− 438	0.83	348	0.33	69	0.70	157	0.12	− 88	0.00	− 65	0.01
70 +		113	0.96	211	0.63	128	0.58	20	0.86	− 66	0.08	− 35	0.27
**Length of stay (LOS)** [Care within 1 day]													
1–7 days*				4544	0.00	4950	0.00	973	0.00	900	0.00	1004	0.00
8–30 days		17,423	0.00	23,953	0.00	24,332	0.00	822	0.00	609	0.00	724	0.00
31–90 days		57,198	0.00	67,098	0.00	66,698	0.00	656	0.01	536	0.00	602	0.00
91–365 days		148,820	0.00	156,946	0.00	152,133	0.00	827	0.02	485	0.00	482	0.00
**Number of planned consultations**		− 24	0.12	− 17	0.00	− 6	0.00	− 1	0.05	− 1	0.00	− 1	0.00
**First hospital visit** [Not a first visit]		− 232	0.95	− 33	0.94	406	0.02	− 92	0.40	43	0.29	83	0.00
**Interval since last hospital episode** [7–30 days]													
Very short re-visit (-3 days)		2197	0.21	2899	0.00	2126	0.00	264	0.00	326	0.00	309	0.00
3–7 days		1069	0.54	369	0.10	447	0.00	28	0.62	39	0.04	68	0.00
30–90 days		1542	0.40	− 48	0.85	26	0.79	− 69	0.27	− 33	0.12	− 3	0.81
90 days -		2255	0.25	101	0.74	250	0.06	− 184	0.01	− 14	0.58	35	0.05
No previous history or anonymous		3769	0.32	− 399	0.31	− 132	0.31	− 125	0.20	− 34	0.30	− 16	0.37
**Admission type**, N (%) [Planned]													
Acute		2782	0.02	− 1075	0.00	− 722	0.00	− 126	0.03	70	0.00	36	0.07
**Care type**, N (%) [Outpatient consultation]													
Ambulatory outpatient consultation				− 685	0.06	− 1343	0.00	− 10	0.93	− 75	0.04	− 230	0.00
Ambulatory other services				− 1203	0.00	− 1531	0.00	− 50	0.67	− 180	0.00	− 314	0.00
Inpatient*				88	0.94	− 645	0.14	− 927	0.00	− 617	0.00	− 342	0.00
Ambulatory inpatient		14,667	0.00	3868	0.01	1445	0.04	− 850	0.00	− 537	0.00	− 262	0.00
Surgical intervention		9094	0.00	33,089	0.02	572	0.32	− 812	0.00	− 512	0.00	− 258	0.00
**Diagnose**, N (%) [Depressive disorder]													
Emotional				454	0.44	841	0.00	− 13	0.97	81	0.09	145	0.00
Trauma				279	0.71	101	0.62	− 407	0.29	21	0.73	24	0.37
Anxiety				1918	0.00	1208	0.00	181	0.61	261	0.00	208	0.00
Schizophrenia		9832	0.02	2934	0.00	2816	0.00	− 269	0.43	283	0.00	413	0.00
Other psychoses		978	0.77	1402	0.02	1678	0.00	− 72	0.84	170	0.00	252	0.00
Bipolar		3437	0.36	1760	0.01	1131	0.00	− 229	0.52	172	0.00	158	0.00
ADHD				− 454	0.61	344	0.19	− 494	0.47	− 27	0.71	53	0.12
Geriatric				2107	0.05	853	0.01	1122	0.04	340	0.00	169	0.00
Eating disorder		7057	0.31	3126	0.01	1522	0.00	58	0.89	233	0.01	228	0.00
Personality disorder		2545	0.70	1290	0.09	1394	0.00	− 160	0.66	118	0.06	195	0.00
OCD				1501	0.09	2759	0.00	318	0.41	258	0.00	466	0.00
Substance abuse		7102	0.04	1413	0.02	1160	0.00	− 228	0.52	184	0.00	200	0.00
Development disorder				− 730	0.67	1097	0.10			− 1	1.00	209	0.03
Stress		− 2086	0.56	1418	0.03	1744	0.00	237	0.50	433	0.00	468	0.00
Organic disorder		2020	0.83	5778	0.02	3514	0.01	− 31	0.94	721	0.00	547	0.00
Neuropsychiatric		40,369	0.03	6572	0.05	4123	0.03			106	0.60	215	0.26
Not specified		− 337	0.94	2706	0.00	1848	0.00	73	0.83	342	0.00	308	0.00
Anonymous		2811	0.37	1869	0.00	1851	0.00	− 79	0.82	257	0.00	314	0.00

* Refencese for 1% analysis

Ambulatory inpatient service was found to be an important variable for all the subgroups, as was a surgical intervention in the 1% and 5% groups. Among the DRGs, schizophrenia and substance abuse were associated with increased total costs in all three high-cost subgroups.

## Discussion

### Cost structure

One of the distinctive features of the cost structure of specialist mental healthcare was that only 1% episodes consumed more than half of total hospital resources. This finding is consistent with the well-known fact that a small proportion of the population consumes a disproportionately large share of healthcare spending [21, 35–37]. Frequent hospital visits are an expected part of the disease pathways of patients with ongoing or life-long functional impairment due to psychiatric disorders. The disproportionate use of health services in a need-based healthcare system and the high costs attributable to a small number of patients are, therefore, unavoidable and rather reasonable. Moreover, a minority subgroup of patients in specialist mental healthcare are forensic patients on court-mandated hospital stays of long duration. The reason for examining particularly expensive episodes, however, is to understand the characteristics of heavy users, to investigate how they utilize the service, and to identify improvement areas for preventing the revolving door phenomenon, when it is possible to do so. High-cost episodes may also be associated with low productivity and substandard service quality, such as multiple avoidable acute readmissions. Focused care for high-cost cases has been implemented in a variety of clinical settings because even small changes can have a significant impact on patient outcomes and healthcare costs. These efforts, however, have not always been successful. For example, the author of a longitudinal study of heavy service users in Switzerland concluded that “preventive interventions to curb excessive service use appear to be out of reach for the majority of heavy users” due to difficulty predicting hospital resource use [38]. Similarly, a study in the United Kingdom reported that a new intervention for heavy users had no discernible effect [39]. However, past studies have calculated hospital resource use based on written questionnaires (e.g., “What inpatient services have you used in the past three months?”) [40] or days of hospitalization; thus it is important to note that improved cost data, such as the data that is the basis for our study, may enhance the precision of analyses. Fortunately, contrary findings have recently demonstrated that some intensive interventions are effective among high-cost patients. For instance, assertive community treatment (ACT), an intensive type of care for people with severe mental disorders, has been demonstrated to have positive outcomes in numerous nations [41].

Other features that stood out in our analysis of the cost structure was the high ratio of labor costs, and the high ratio of inpatient costs in the expensive groups. The proportion of labor costs in specialist mental healthcare seems to have been stable for a long time. According to Finnish hospital cost research in 1980, the cost of labor accounted for 87% of the total cost [42], which is identical to the 87% found in our 2018–2021 data. This implies that the minimum number of personnel required to care for psychiatric patients has remained constant over time, and that technological investment in mental health settings has been low. A high ratio of inpatient care costs for expensive groups was also reported in other studies [27, 43].

We stratified the costs for the 1%, 5%, and 10% most expensive ‘individual patient’ (not episodes) to make further comparisons with previous studies. As mental health patients typically have multiple episodes with various diagnoses, we summed the total amount of cost spent per patient. The results were remarkably consistent with those of a US study that covered the time period from 1920 to the 1980s [44] and a systematic review that covered references from 1995 to 2012 [21] (see Appendix 4). The striking similarity of the resource-use concentration and structures across time and country suggests that the existing findings of high-cost patients and heavy user research may be useful in the Norwegian context. For instance, a systematic review of studies of high-cost patients estimated that a maximum of 10% of the total cost was considered a preventable expenditure [21].

### Cost drivers

LOS had the greatest impact on hospital resource utilization. It is not surprising that the care costs rise as the number of days spent in the hospital increases. However, it was unclear whether prolonged hospital stays themselves would exacerbate the increase in total costs. The “average cost per day,” which removes the cumulative effect of LOS, tended to decrease as LOS increased. The average care cost was highest on the first seven days (see Appendix 5). Cromwell et al. reported comparable results for hospitalization costs and LOS [45]. According to that study, the first day of hospitalization was expensive due to the auxiliary services provided at the time of admission. Prolonged hospitalization was the biggest reason for the cost increase, but the rate of cost growth slowed down as hospital LOS increased, possibly reflecting the cost-reducing effect of long-term hospitalization. These findings are consistent with those of early US efforts to reduce LOS in order to contain medical expenses [46]. However, some studies have revealed the possibility that LOS reduction may not be an effective way to curb total costs due to the relatively low marginal cost of last-day-stay (the final phase of hospitalization is primarily for recuperation) [47], and a possible trade-off effect between a shorter LOS and increased readmissions [48]. Efforts to reduce LOS in Norway were accelerated by the implementation of the Norwegian Coordination Reform in 2012 [49]. The LOS In psychiatry has decreased by approximately 30% compared to 2009, which means it decreased from an average of 27 days to 18 days [50]. No research has yet been conducted in Norway on the effects of a shorter psychiatric LOS on total healthcare costs; however, a nationwide post-reform qualitative study suggests that it is unclear whether the shorter LOS resulted in a decrease in overall healthcare costs due to a marked increase in reported hospital readmissions [51]. In Norwegian context, this may suggest that a reduction in hospital bed-days may increase municipal expenditures. Based on these facts, the findings of this study suggest that a reduction in LOS in specialist mental healthcare may not always be associated with a cost reduction, even though LOS is the most influential factor that drives total costs. This result has potential implications for policymakers and clinicians collaborating with patients in discharge processes.

First hospital visit refers to the initial visit registered during the follow-up period of 2018–2021. Although this variable was not sufficient to account for extremely expensive episodes of 1% or 5%, it appears that first hospital visits use more resources than subsequent visits. Along with the results obtained from the LOS analysis, a possible implication is that enhancing the initial experience of patients, such as logistics optimization and better use of capacity, can also be economically beneficial for hospitals.

The interval between two episodes was a very interesting predictor in this data. Overall, these results indicate that costs are more likely to deviate from the mean when the intervals between visits are extremely short or extremely long. This suggests two prototypical clinical scenarios: first, a discharge that is too early while the patient remains acutely ill, leading to rapid readmission; and second, chronic and episodic pathology, which requires more serious evaluation and treatment upon readmission. The first of these may be preventable through better policy; the second may not be. Because the present data is limited to a four-year time frame, longer intervals were excluded, which could result in an underestimation of the number of people and typical time between visits. Consequently, the actual effect should be expected to be higher than the observed effect.

The current study found that one additional outpatient consultation added approximately USD 363 to the total cost (see Appendix 6). However, as the number of planned consultations increased, there was a very small but statistically significant total cost reduction. This result can be interpreted in two ways. Patients who receive frequent outpatient consultations have a lower overall cost; put differently, healthier patients utilize psychosocial consultations more often. Alternately, increased psychiatric/psychotherapeutic consultations have a long-term cost-saving effect that can offset the cost of added consultations. Many studies have demonstrated there is an economic value of psychotherapy via a reduction in other medical and/or social costs. For example, more than one-third of hospitalized patients for medical and surgical reasons have psychiatric comorbidities [52, 53], and appropriate psychiatric interventions can decrease the LOS of these patients, thereby reducing total costs [54, 55]. This is one of the most studied effects used to demonstrate the economic value of psychotherapy. Our data provide intriguing empirical evidence that psychotherapeutic consultation may already have a cost-offsetting effect in specialist mental healthcare, mitigating high costs by preventing patient deterioration over the long term. Helse Førde is known to have relatively lower outpatient consultation rate than the national average [56–59]. Therefore, generalizations require caution: cost-offsetting effect of psychotherapeutic consultations might be underestimated.

Surgery had a significant effect on increasing the total cost of all the expensive subgroups, although only 133 episodes out of 180,220 records included surgical interventions (Table 1), and the cost of the operation and anesthesia itself was negligible, accounting for only 0.1% of the total cost (Table 2). Surgical intervention is a reliable indicator of high resource consumption because it may indicate severe depression, mood disorders and self-harm. The effect of surgical intervention is reflected in the costs of other hospital activities, such as intensive care, radiology, and wards, resulting in an increase in total costs.

DRGs had a relatively minor impact on costs compared to LOS and care type, yielding inconsistent outcomes across groups. Among expensive episodes, schizophrenia and substance abuse were associated with increased total costs in all the expensive subgroups. Cost studies on mental disorders have produced similar findings for schizophrenia [1, 28, 60, 61], substance use [1, 61–64], and organic disorders [61]; (including dementia). As the treatment cost for dementia increases with symptoms [65] and disease progression [66], keeping patients at an earlier stage is beneficial for both the patient and the healthcare system. This also applies to schizophrenia. Relapse prevention has been shown to be crucial for controlling healthcare costs in many studies [67–69].

Our study revealed that male patients and younger patients had higher costs; however, the associations of age and gender with high-cost episodes were not consistent. Several studies have reported opposite results for females and older age groups [1, 27, 70] while others have reported the same association between young age and high hospital costs [61, 71–73]. Gender and age variables appear to have a range of results depending on the research data and patient episode mix.

As seen in Table 1, there was a smaller proportion of males involved in overall episodes, but a higher proportion of males involved in expensive episodes, which may indicate males exhibit a low level of help seeking behavior, which is well-documented in psychiatry [74, 75]. Male patients accounted for 44% of hospital visits, but 50–56% of the expensive episodes in our study. This might also reflect the fact that the Norwegian mental healthcare system treats significantly more men than women who have been sentenced by the court to involuntary psychiatric treatment after committing violent crimes. Such admissions are often lengthy and resource intensive.

Similarly, the elderly population (over 60 years old) accounted for only 5% of all hospital visits, but between 7 and 20% of the expensive episodes. Given that successful out-patient consultation could lead to less in-patient bed usage [76], efforts to engage these patients in regular mental healthcare services prior to the onset of severe episodes may be economically beneficial to the hospital, as well as advantageous to patients if they are satisfied with this level of care.

This is the first study in Norway to explore the individual-level cost structure of specialist mental healthcare. Our research provides a review of recent patterns of psychiatric patient resource utilization, cost-increasing factors, and differences between domestic and international findings. The current study covers a comprehensive number of episodes, including child and adolescent patients, and includes various types of disease pathways. Hence, the problem of selection bias is not very likely. In addition, by using a four-year time horizon, we analyzed the results for longer-term resource utilization patterns compared to previous LOS and cost studies.

This research has some limitations. Its biggest potential weakness is the anonymous groups in DRG. This was necessary due to the need to protect personal information, but this may have impaired the accuracy of the DRG-related analyses. Particularly, adolescence (age group 10–19) was a significant factor that increased the cost of 1% episodes, but the DRGs of nearly 47% of the episodes in the adolescence group were anonymized. Although these diagnoses are rare and affect a small number of patients, they account for a significant portion of hospital resource utilization. This suggests that appropriate research and special treatment training for clinicians can have a positive economic impact on the healthcare system. Another potential weakness is that our data were limited to costs of special mental healthcare divisions, which may underestimate the true costs associated with high-cost psychiatric patients. A growing body of research demonstrates that high-cost patients with mental illness consume more hospital resources than do non-psychiatric high-cost patients [77]. Recent meta-analyses suggest that patients with severe mental illness make greater use of non-psychiatric health services and represent a greater economic burden on hospitals [78]. Similar results have been reported for children and adolescents [79]. A comprehensive study incorporating relevant somatic costs could help estimate the true impact of heavy-use in specialist mental healthcare.

## Conclusion

A specialist mental healthcare division has a unique cost structure. As 1% of episodes consumed 57% of hospital resources, any intervention that effectively prevents high-cost episodes will be likely to result in significant resource savings. LOS is the most reliable and influential factor in hospital resource consumption. However, if a shorter LOS results in frequent readmissions, it would be counterproductive in the long-run, as the costs incurred on the early days of readmission are likely to be higher than the costs incurred on the later days of previous admission. Our study revealed that first hospital visits and too short re-visits are more expensive than other visits. Therefore, improving the initial flow of hospital care, where resource utilization is intensive, could significantly enhance cost-efficiency. Our study found empirical evidence that total resource consumption is likely to decrease as the number of planned outpatient consultations increases, in terms of total costs over a relatively long period of four-years. Due to the small magnitude of the effect, however, additional research is needed. Finally, male and elderly populations in the region have been observed to make fewer hospital visits, but they account for a higher proportion of expensive subgroups. Efforts to help these patients have a stable connection with a care provider before they have expensive and serious episodes might be clinically and financially beneficial. These findings should be incorporated into future healthcare policies to improve patient care and optimize hospital resource utilization.

## Figures and Tables

**Fig. 3:**
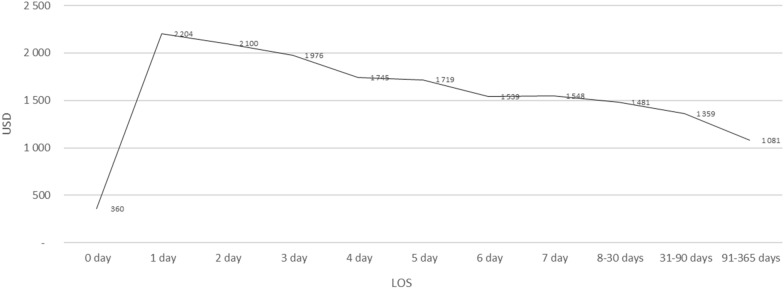
Average treatment cost per LOS (USD)

## Data Availability

In line with the requirements of the data protection officer of Førde Hospital Trust, reasonable requests for access to data are to be made in writing to the corresponding author.

## Appendix 1

See Table 5

**Table 5:** Detailed cost categories of the KPP according to traditional and activity-based perspectives

Perspective	Structure level1	Structure level2	Structure level3	Structure level4
Traditional perspective	Direct costs	Direct labor costs	Ambulatory service	Personnel
			Anesthesia	Personnel
			Intensive care	Personnel
			Medicines Direct	Personnel
			Operation	Personnel
			Outpatient	Personnel
			Radiology	Personnel
			Inpatient care	Personnel
		Non-labor costs	Ambulatory service	Consumables
			Anesthesia	Consumables
			Intensive care	Consumables
			Medicines Direct	Consumables
			Operation	Consumables
			Outpatient	Consumables
			Radiology	Consumables
			Inpatient care	Consumables
			Ambulatory service	Other consumable
			Anesthesia	Other consumable
			Intensive care	Other consumable
			Medicines Direct	Other consumable
			Operation	Other consumable
			Outpatient	Other consumable
			Radiology	Other consumable
			Inpatient care	Other consumable
			Ambulatory service	Implants
			Anesthesia	Implants
			Intensive care	Implants
			Medicines Direct	Implants
			Operation	Implants
			Outpatient	Implants
			Radiology	Implants
			Inpatient care	Implants
			Ambulatory service	Medicines
			Anesthesia	Medicines
			Intensive care	Medicines
			Medicines Direct	Medicines
			Operation	Medicines
			Outpatient	Medicines
			Radiology	Medicines
			Inpatient care	Medicines
	Indirect costs		Ambulatory service	Other cost
			Anesthesia	Other cost
			Intensive care	Other cost
			Medicines Direct	Other cost
			Operation	Other cost
			Outpatient	Other cost
			Radiology	Other cost
			Inpatient care	Other cost
			Ambulatory service	Capital cost
			Anesthesia	Capital cost
			Intensive care	Capital cost
			Medicines Direct	Capital cost
			Operation	Capital cost
			Outpatient	Capital cost
			Radiology	Capital cost
			Inpatient care	Capital cost
			Ambulatory service	Cost reduction
			Anesthesia	Cost reduction
			Intensive care	Cost reduction
			Medicines Direct	Cost reduction
			Operation	Cost reduction
			Outpatient	Cost reduction
			Radiology	Cost reduction
			Inpatient care	Cost reduction

Perspective	Structure level1	Structure level2
Activity-based perspective	Ambulatory service	Other consumable
		Consumables
		Implants
		Capital cost
		Cost reduction
		Medicines
		Other cost
		Personnel
	Anesthesia	Other consumable
		Consumables
		Implants
		Capital cost
		Cost reduction
		Medicines
		Other cost
		Personnel
	Intensive care	Other consumable
		Consumables
		Capital cost
		Cost reduction
		Medicines
		Other cost
		Personnel
	Operation	Other consumable
		Consumables
		Implants
		Capital cost
		Cost reduction
		Medicines
		Other cost
		Personnel
	Outpatient	Other consumable
		Consumables
		Implants
		Capital cost
		Cost reduction
		Medicines
		Other cost
		Personnel
	Radiology	Other consumable
		Consumables
		Implants
		Capital cost
		Cost reduction
		Medicines
		Other cost
		Personnel
	Inpatient care	Other consumable
		Consumables
		Implants
		Capital cost
		Cost reduction
		Medicines
		Other cost
		Personnel

Consumables category includes the cost of medicines provided to patients in connection with patient contact. They are classified separately from the Medicine category for administrative purposes. Other consumables category have been created as a collective category for all other consumables used in patient care. Other cost category is set up for reflecting actual costs that are allocated to the patients such as cost regards unmet schedule or empty beds. More information can be found on < Revidert versjon Nasjonal spesifikasjon for KPP-modellering—psykisk helsevern og TSB (Begreper og metoder) >

## Appendix 2

See Table 6

**Table 6:** DRG matching list

Original DRG code	DRG Code name	Categories
TD02A	Grupperettede tiltak—Andre grupperettede tiltak—Voksne	Not specified
TD02B	Grupperettede tiltak—Andre grupperettede tiltak—Barn og unge	Not specified
TD03A	Familierettede polikliniske tilbud—Voksne	Not specified
TD03B	Familierettede polikliniske tilbud—Barn og unge	Not specified
TD10A	Polikliniske konsultasjoner—Alkoholavhengighet—Voksne	Substance abuse
TD11A	Polikliniske konsultasjoner—Opioidavhengighet—Voksne	Substance abuse
TD12A	Polikliniske konsultasjoner—Cannabisavhengighet—Voksne	Substance abuse
TD18A	Polikliniske konsultasjoner—Samtidig rusproblem og alvorlig psykisk lidelse—Voksne	Substance abuse & APL/SMI
TD19A	Polikliniske konsultasjoner—Annen rusmiddelavhengighet—Voksne	Substance abuse
TD20A	Polikliniske konsultasjoner—Schizofreni—Voksne	Schizophrenia
TD21A	Polikliniske konsultasjoner—Andre psykoser—Voksne	Other psychoses
TD31A	Polikliniske konsultasjoner—Bipolar lidelse—Voksne	Bipolar
TD32A	Polikliniske konsultasjoner—Andre depressive tilstander—Voksne	Depressive disorder
TD32B	Polikliniske konsultasjoner—Depresjon—Barn og unge	Depressive disorder
TD33A	Polikliniske konsultasjoner—Alvorlig depresjon—Voksne	Depressive disorder
TD38A	Polikliniske konsultasjoner—Emosjonelle symptomer og tegn—Voksne	Emotional
TD38B	Polikliniske konsultasjoner—Emosjonelle symptomer og tegn—Barn og unge	Emotional
TD40A	Polikliniske konsultasjoner—Angst og fobiske lidelser—Voksne	Anxiety
TD40B	Polikliniske konsultasjoner—Angst og fobiske lidelser—Barn og unge	Anxiety
TD426A	Innleggelser—Bipolare lidelser < 60 år	Bipolar
TD426B	Innleggelser—Bipolare lidelser > 59 år	Bipolar
TD426C	Innleggelser—Andre affektive lidelser < 60 år	Other psychoses
TD426D	Innleggelser—Andre affektive lidelser > 59 år	Other psychoses
TD427A	Innleggelser—Angstlidelse	Anxiety
TD427B	Innleggelser—Vedvarende belastnings- og tilpasningsforstyrrelser	Stress
TD427C	Innleggelser—Akutt stressreaksjon	Stress
TD428N	Innleggelser—Personlighetsforstyrrelser	Personality disorder
TD429B	Innleggelser—Organiske betingede mentale forstyrrelser u/bk	Organic disorder
TD42A	Polikliniske konsultasjoner—Tvangslidelser—Voksne	OCD
TD42B	Polikliniske konsultasjoner—Tvangslidelser—Barn og unge	OCD
TD430A	Innleggelser—Schizofreni < 30 år	Schizophrenia
TD430B	Innleggelser—Schizofreni 30–59 år	Schizophrenia
TD430C	Innleggelser—Schizofreni > 59 år	Schizophrenia
TD430D	Innleggelser—Andre langvarige psykoselidelser	Other psychoses
TD431C	Innleggelser—Andre mentale forstyrrelser hos barn	Not specified
TD432C	Innleggelser—Andre innleggelser relatert til tilstander i HDG 19	Not specified
TD436A	Innleggelser—Tilstander relatert til rusmiddelmisbruk m/bk	Substance abuse
TD436B	Innleggelser—Tilstander relatert til rusmiddelmisbruk u/bk	Substance abuse
TD436C	Innleggelser—Rusutløst psykose	Substance abuse
TD43A	Polikliniske konsultasjoner—PTSD og tilpasningsfrostyrrelser m.v.—Voksne	Trauma
TD43B	Polikliniske konsultasjoner—PTSD og tilpasningsfrostyrrelser m.v—Barn og unge	Trauma
TD499	Innleggelser—Somatiske og andre tilstander utenom HDG 19	Not specified
TD60A	Polikliniske konsultasjoner—Personlighetsforstyrrelser—Voksne	Personality disorder
TD71A	Annen omfattende testing og kartlegging med bruk av standardsierte verktøy—Voksne	Not specified
TD71B	Annen omfattende testing og kartlegging med bruk av standardsierte verktøy—Barn og unge	Not specified
TD72B	Observasjonstiltak i skole og barnehage m.v.—Barn og unge	Not specified
TD802A	Terapeutiske, strukturerte polikliniske dagtilbud for psykiske og rusrelaterte lidelser—Voksne	Not specified
TD802B	Terapeutiske, strukturerte polikliniske dagtilbud for psykiske og rusrelaterte lidelser—Barn og unge	Not specified
TD80A	Polikliniske konsultasjoner—Alderspsykiatriske problemstillinger—Voksne	Geriatric
TD81A	Polikliniske konsultasjoner -Spiseforstyrrelser—Voksne	Eating disorder
TD81B	Polikliniske konsultasjoner—Spiseforstyrrelser—Barn og unge	Eating disorder
TD84B	Polikliniske konsultasjoner—Autisme og andre gjennomgripende utviklingsforstyrrelser—Barn og unge	Development disorder
TD90A	Polikliniske konsultasjoner—ADHD o.l.—Voksne	ADHD
TD90B	Polikliniske konsultasjoner—ADHD o.l.—Barn og unge	ADHD
TD91B	Polikliniske konsultasjoner—Barn under 5 år	Not specified
TD93A	Samarbeid- og oppfølgingsmøte med samarbeidspart utenfor spesialisthelsetjenesten—Voksne	Not specified
TD93B	Samarbeid- og oppfølgingsmøte med samarbeidspart utenfor spesialisthelsetjenesten—Barn og unge	Not specified
TD94A	Telefonkonsultasjon for psykiske eller rus- og avhengighetsrelaterte problemstillinger—Voksne	Not specified
TD94B	Telefonkonsultasjon for psykiske eller rus- og avhengighetsrelaterte problemstillinger—Barn og unge	Not specified
TD95A	Oppfølgingssamtale per telefon med samarbeidspart utenfor spesialisthelsetjenesten—Voksne	Not specified
TD95B	Oppfølgingssamtale per telefon med samarbeidspart utenfor spesialisthelsetjenesten—Barn og unge	Not specified
TD96A	Konsultasjon med pårørende—Voksne	Not specified
TD96B	Konsultasjon med foresatte/pårørende—Barn og unge	Not specified
TD97	Poliklinisk fysisk trening som ledd i spesialisthelsetjenester til pasienter med psykiske eller rusrelaterte lidelser	Not specified
TD981	Innleggelser uten overnatting—Psykiske og rusrelaterte tilstander	Not specified
TD99A	Andre polikliniske konsultasjoner innen PHV&TSB—Voksne	Not specified
TD99B	Andre polikliniske konsultasjoner innen PHV&TSB—Barn og unge	Not specified
XD90A	Polikliniske konsultasjoner—Andre problemstillinger—Voksne	Not specified
XD90B	Polikliniske konsultasjoner—Andre problemstillinger—Barn og unge	Not specified
TD432A	Innleggelser—Spiseforstyrrelser	Eating disorder
TD99L	Poliklinisk oppmøte for utlevering eller administrasjon av LARlegemiddel	Not specified
TD801A	Diagnostiske, strukturerte polikliniske dagtilbud for psykiske og rusrelaterte lidelser—Voksne	Not specified
TD431B	Innleggelser—Nevropsykiatriske forstyrrelser	Neuropsychiatric

## Appendix 3

See Table 7

**Table 7:** Comparison with Norwegian national data (SAMDATA, 2021)

(USD)						
Group categories		SAMDATA (Norway average)				Current data
		2018	2019	2020	2021	2018–2021
Cost per outpatient consultation	All	345	330	355	350	363
Cost per bed-day	Adult	1384	1396	1482	1462	1259
	TBS	888	926	995	996	
	Children and adolescent	2070	2553	2439	2374	2261
Cost per patient	Adult	11,782	11,593	11,770	11,525	18,152**
	TBS	11,737	11,063	11,252	11,677	
	Children and adolescent	7902	8021	8045	7621	

* Differences in criteria for the children and adolescent groups: SAMDATA (age 0–18), current KPP data (age 0–19)

** In the four-year base calculations, the number of overlapping patients per year was consolidated, resulting in a reduction of the denominator. The annual 'cost per patient' of current data is: USD 9205 (2018), USD 10,051 (2019) USD 10,332 (2020), USD 8719 (2021)

## Appendix 4

See Table 8

**Table 8:** Distribution of health expenditures for patient in the 1%, 5%, and 10% groups

Category	Distribution of health expenditures								Current Data
	Berk, M. L., & Monheit, A. C. (2001) [28]							Wammes, J.J.G. et al. [11]	
Year	1928	1963	1970	1977	1980	1987	1996	1995–2021 average	2022
1%	–	17%	26%	27%	29%	28%	27%	24%	24%
5%	52%	43%	50%	55%	55%	56%	55%	55%	55%
10%	–	59%	66%	70%	70%	70%	69%	68%	70%

## Appendix 5

See Fig. 3

## Appendix 6

See Table 9

**Table 9:** Average treatment cost per DRG for inpatient, outpatients, and total patients

DRG category	Average price per episode			(USD)
		Outpatient	Inpatient	
				Mean LOS
Emotional	369	369	–	–
Depressive disorder	326	326	–	–
Trauma	336	336	–	–
Anxiety	558	339	25,114	16
Schizophrenia	3087	571	47,219	38
Other psychoses	8872	433	32,672	25
Bipolar	1800	350	32,481	25
ADHD	331	331	–	–
Geriatric	360	360	–	–
Eating disorder	858	312	56,575	27
Personality disorder	811	346	12,121	10
OCD	450	450	–	–
Substance abuse	4861	343	39,663	31
Development disorder	362	362	–	–
Stress	19,619	–	19,619	13
Organic disorder	16,238	–	16,238	9
Neuropsychiatric	93,811	–	93,811	62
Average	9003	363	32,304	–

* Total cost/number of episodes

## Abbreviations

AIC: Akaike information criterion
ACT: Assertive community treatment
BIC: Bayesian information criterion
CPP: Cost per patient
DRG: Diagnosis-related group
FACT: Flexible assertive community treatment
GLM: Generalized linear model
KPP: Kostnad per pasient (En: Cost per patient)
LOS: Length of stay
NOK: Norwegian kroner
